# The Canadian alcopop tragedy should trigger evidence‐informed revisions of federal alcohol regulations

**DOI:** 10.1111/dar.12896

**Published:** 2019-02-04

**Authors:** Catherine Paradis, Nicole April, Claude Cyr, Réal Morin, Manon Niquette

**Affiliations:** ^1^ Canadian Centre on Substance Use and Addiction Ottawa Canada; ^2^ Institut National de Santé Publique du Québec Québec Canada; ^3^ Pediatrician Centre Hospitalier Universitaire de Sherbrooke Sherbrooke Canada; ^4^ Vice‐Présidence aux Affaires Scientifiques Institut National de Santé Publique du Québec Québec Canada; ^5^ Department of Information and Communication, Université Laval Québec Canada

**Keywords:** Canada, government regulation, alcoholic intoxication, alcoholic beverages

## Abstract

On 1 March 2018, a 14‐year‐old girl was found lifeless in a stream behind her high school after having consumed FCKDUP—a beverage containing 11.9% alcohol and sold in 568 mL cans—during her lunch hour. Following her death, the Canadian government took actions at ministerial and parliamentary levels by seeking experts’ advice to better regulate highly sweetened alcoholic beverages, otherwise referred to as ‘alcopops’. We suggest that the Canadian government uses the work surrounding the alcopop tragedy as an opportunity to make significant amendments and revisions of federal alcohol regulations.

On 1 March 2018, in the Canadian town of Laval, a 14‐year‐old girl was found lifeless in a stream behind her high school, after having consumed FCKDUP—a beverage containing 11.9% alcohol, guarana (a natural stimulant that contains caffeine), a large amount of sugar and sold in 568 mL cans for approximately 3.33 CAD—during her lunch hour. Sadly, the death occurred in the midst of a series of media and scientific reports all pointing to the danger of highly sweetened alcoholic beverages, otherwise referred to as ‘alcopops’. Following the young girl's death, the Canadian government took actions at the ministerial and parliamentary levels.

On 19 March 2018, the House of Commons Standing Committee on Health (HESA) adopted a motion to undertake an urgent study ‘to develop recommendations on actions that the federal government could take, in partnership with the provinces and territories, to better regulate pre‐mixed drinks combining high alcohol, caffeine and sugar content’ 1. HESA announced it would report its findings and recommendations to the House no later than June 2018, and that it would request that the government table a comprehensive response to the report.

On 28 March 2018, Health Canada issued a ‘Notice of intent to amend the Food and Drug Regulations to restrict the amount of alcohol in single‐serve highly sweetened alcoholic beverages’ to reduce the health and safety risks of these products 2. The notice announced that Health Canada was consulting on the mechanism by which to restrict the amount of alcohol in a single‐serve container and on the sweetness threshold that would trigger the restrictions. It further announced it would convene a meeting with provincial and territorial representatives and key stakeholders on 8 June 2018, to discuss collective measures for reducing the risks of alcopops.

Consultations gave experts the opportunity to highlight a worrying trend toward an increasing number of young Canadians being hospitalised for acute alcohol poisoning. The Institut national de santé publique du Québec shared a report showing that the rate of acute alcohol poisoning in the province of Quebec was 365 cases per 100 000 among people at ages 18–19 years 3. Others noted that 55% of undergraduate students report harm when they drink alcohol 4. In one Canadian campus towns, at least three youths were taken to the local hospital emergency department every week for an alcohol‐related medical emergency and among that group 25% of them had their lives in danger upon arrival 5.

Throughout consultations at both the ministerial and parliamentary levels, experts identified ways of reducing the health and safety risks posed by alcoholic beverages in general and alcopops in particular. Experts recommended limiting alcopops to one standard serving of alcohol per container, that is, 13.6 g of pure alcohol, introducing labelling requirements and rotating warning labels for alcoholic beverages and addressing taxation and pricing incentives with a priority on setting a minimum price for all alcoholic beverages, adjusted according to alcohol content 6, 7. Communication experts detailed the extent to which alcopop companies use social media strategies that appeal specifically to young people and argued that it is necessary to place new restrictions on the marketing of these products 3. Overall, the experts put forward the World Health Organization's ‘best‐buys’ for reducing the harmful use of alcohol 8.

At the same time that Health Canada was collecting evidence to make recommendations about the *Food and Drug Act*, the alcohol industry was actively lobbying the government. Public records from the Office of the Commissioner of Lobbying show that between 11 April and 26 September 2018, registered lobbyists working on behalf of the Geloso Group, maker of FCKDUP, communicated 18 times with 60 government representatives, including the Minister of Health, the Minister of Finance, the Minister of Public Safety Canada, the Minister of Transport, members of parliament and Senior Advisors to the Prime Minister's Office 9. Records indicate that the government was lobbied to ‘update highly sweetened alcoholic beverages regulation by encouraging the federal government to find a comprehensive solution to the risks associated with alcohol consumption among minors and to restrict minors’ access to these products’. Yet, the brief submitted by the Geloso Group to the HESA committee was rather designed to emphasize that the Group, ‘of its own initiative and even before Quebec and Canada's governments intervened, ordered the withdrawal of its product’ 10. Because of this pre‐emptive action, many outside commentators, including the authors of this commentary, wonder if the Geloso Group's real intentions were never to encourage the federal government to find a comprehensive solution, but rather to convince the government that amendments to the *Food and Drug Act* are not necessary. The alcohol industry has in the past opposed regulation by emphasising industry responsibility and the effectiveness of self‐regulation 11, 12, 13, 14.

At the time of the writing, a few weeks away from Health Canada releasing its proposed regulations, there are concerns that there will not be significant amendments to the *Food and Drug Regulations.* However, for those of us who have been following this case, it will be difficult to forget what the ministerial and the parliamentary consultations have clearly brought to light: Athena Gervais died after consuming a drink that was readily available, cheap and marketed toward youth.

We suggest that Health Canada uses the work surrounding the alcopop tragedy as an opportunity to make significant amendments and revisions to all federal alcohol‐related regulations, including labelling and packaging regulations, excise tax policies and advertising control policies. It is encouraging that on 5 September 2018, Health Canada announced its decision to consult Canadians on how to strengthen the federal government's health‐focused approach to substance use issues and its wish to update the Canadian Drugs and Substances Strategy to more effectively and compassionately address the use of psychoactive substances, including alcohol, in Canada 15. However, the real test will be to see how Health Canada will turn the results of all these consultations into action. With an overall view to avoid another tragedy and reduce alcohol‐related harm, we strongly encourage Health Canada to prioritise alcohol policy in general and implement the most cost‐effective interventions.
